# Music Therapy Modulates Abnormal Brain Networks and Alleviates Anxiety Symptoms in University Students: An fNIRS Study

**DOI:** 10.1155/da/7906429

**Published:** 2025-11-20

**Authors:** Lijuan Liang, Yue Zhu, Zhiguo Zheng, Hui Ma, Xuefeng Cai, Hua Yang, Fei Wang

**Affiliations:** ^1^Department of Psychology, The First Affiliated Hospital of Hainan Medical University, Haikou, China; ^2^Early Intervention Unit, Department of Psychiatry, Affiliated Nanjing Brain Hospital, Nanjing Medical University, Nanjing, China; ^3^School of Information Engineering, Hainan Vocational University of Science and Technology, Haikou, China; ^4^School of Information and Communication Engineering, Hainan University, Haikou, China; ^5^Psychological Counseling and Treatment Center, Hainan Provincial Anning Hospital, Haikou, China

**Keywords:** anxiety, biomarkers, brain connectivity, functional near-infrared spectroscopy (fNIRS), music therapy

## Abstract

Anxiety's prevalence is increasing, making it a widespread mental health concern. However, scale-based diagnostic methods have limitations. Music therapy helps regulate emotions and alleviate anxiety symptoms. Functional near-infrared spectroscopy (fNIRS) offers a novel approach to diagnosing mental disorders by measuring changes in the concentrations of oxygenated hemoglobin (HbO) and deoxygenated hemoglobin (HbR) in the superficial layers of the brain, thereby reflecting brain activation. This is the first study to use fNIRS to examine the impact of music therapy on anxiety. fNIRS was used to measure changes in HbO and HbR in the superficial brain regions of individuals with anxiety symptoms to evaluate music therapy effectiveness and identify brain regions associated with anxiety. This study recruited 83 participants: 17 comprised the healthy control group, and 66 comprised the anxiety group. The anxiety group was divided into an intervention group (34 participants) and a waiting-list group (32 participants). The intervention group underwent 12 music therapy sessions and exhibited significant changes compared with the waiting group. These changes included connectivity between Wernicke's area and the dorsolateral prefrontal cortex (DLPFC) as well as the visual association cortex and Broca's triangular area. These results suggested that the connectivity characteristics of these brain regions were associated with anxiety. Music therapy significantly improved brain network connectivity characteristics in individuals with anxiety symptoms. Furthermore, fNIRS indicators could serve as biomarkers for the auxiliary identification of anxiety symptoms, aiding early identification and intervention.

**Trial Registration:** ClinicalTrials.gov identifier: NCT05648539

## 1. Introduction

### 1.1. Progression of Anxiety

The global prevalence of anxiety is steadily increasing, making them one of the most common mental health issues worldwide. According to World Health Organization data, ~301 million people worldwide are affected by anxiety, accounting for 3.9% of the global population [1]. The prevalence of generalized anxiety disorder (GAD) in China is 4.1% (3.9% in men and 4.3% in women). This disorder may lead to other mood disorders and even suicidal behaviors [2]. GAD is a common anxiety with a disease burden that significantly affects an individual's quality of life. Numerous studies have shown that the negative impact of GAD is comparable to that of major depressive disorder (MDD) [3]. GAD is characterized by generalized and persistent anxiety, often resulting in symptoms such as nervousness, motor tension, and hyperactivity of the autonomic nervous system [4].

### 1.2. Treatment and Intervention Methods for Anxiety

Anxiety symptoms are common psychological disorders that significantly impair the daily lives and social functioning of individuals. With advancements in psychological medicine, treatment options for anxiety have become increasingly diverse, encompassing pharmacological therapy, psychotherapy, physical therapy, alternative therapies, and lifestyle modifications. Selective serotonin reuptake inhibitors (SSRIs) and serotonin-norepinephrine reuptake inhibitors (SNRIs) are considered first-line pharmacological treatments for GAD, panic disorder (PD), and social anxiety disorder (SAD) [5, 6]. Benzodiazepines are also commonly used, although they carry a risk of dependence [7]. Pharmacological treatments typically require several weeks to take effect and are often accompanied by dependency risks and various side effects.

Cognitive behavioral therapy (CBT) effectively alleviates anxiety symptoms by addressing avoidance behaviors and cognitive biases, such as correcting erroneous beliefs that interpret benign situations as threatening [8]. In the clinical setting, patients with anxiety undergoing CBT have demonstrated significant improvements, with a considerable proportion reporting a notable reduction in symptoms after multiple treatment sessions [9]. CBT is particularly effective for a range of anxiety, including panic, social anxiety, and generalized anxiety [10]. However, the effectiveness of CBT depends largely on the therapist's expertise and experience. Insufficient therapeutic competence may result in ineffective treatment or cause patients to lose confidence in therapy.

In recent years, music therapy has played a significant role in the treatment of anxiety and has emerged as a promising nonpharmacological intervention. It has demonstrated notable efficacy in various populations, including adolescents, adults, and severe cases [11–13]. Studies have shown that music therapy can regulate neurotransmitter levels, alleviate anxiety symptoms, and enhance emotional well-being. It has been particularly effective in reducing the severity of anxiety symptoms in adolescents and young adults, highlighting its potential as a standalone and adjunctive treatment [14]. Music therapy supports emotional self-regulation and adaptive coping skills that are critical for managing anxiety and other intense emotions in adolescents. By participating in music therapy, adolescents develop adaptive strategies for anger management and emotional regulation, thereby improving psychological functioning [15]. It also reduces anxiety symptoms effectively, providing a valuable complementary treatment for common mental health symptoms among adolescents [16].

### 1.3. Recent Applications of Functional Near-Infrared Spectroscopy (fNIRS) in Anxiety Symptoms

fNIRS is a noninvasive neuroimaging technique that evaluates cortical brain activity by measuring fluctuations in oxygenated (oxy-Hb) and deoxygenated hemoglobin (deoxy-Hb) levels [17]. Recent studies have demonstrated the potential of fNIRS for assessing and monitoring the neural correlates of anxiety and the effectiveness of related treatments. One study employed fNIRS to investigate changes in brain functional connectivity (FC) and the reduction of anxiety symptoms in patients with GAD [18]. Another study found that individuals with anxiety showed significantly decreased FC and network efficiency during task performance compared to healthy controls, especially in those with more severe symptoms [19, 20].

### 1.4. Relationship Between Anxiety and Brain Regions

GAD is closely associated with emotional dysregulation. Individuals with generalized anxiety with GAD exhibit hyperreactivity to emotional stimuli, characterized by excessive bottom-up activation. Studies have shown that patients with GAD display higher connectivity within the executive control network of frontoparietal areas [21]. Research on brain networks under different emotional conditions has revealed that patients with GAD exhibit distinct network connectivity patterns when processing various emotions, showing stronger neural network density in response to negative stimuli and indicating significant abnormalities in top-down processing [22, 23].

The dorsolateral prefrontal cortex (DLPFC) plays a critical role in emotional processing, and the pathological mechanisms of GAD are strongly related to dysfunction of the DLPFC, particularly during emotional regulation. Studies have found significant changes in the concentration of N-acetyl aspartate (a marker of neuronal vitality) in both the left and right DLPFC in patients with GAD [24]. Intervention studies have demonstrated that low-frequency repetitive transcranial magnetic stimulation (rTMS) targeting the right DLPFC significantly improves the clinical symptoms of GAD. EEG studies have shown that low-frequency rTMS stimulation of the right DLPFC alleviates anxiety symptoms and markedly reduces network connectivity in the right DLPFC at different time points [25]. Therefore, the DLPFC is a key region associated with anxiety symptoms, although other brain regions are also involved in emotional regulation processes linked to anxiety [26, 27]

Individuals with higher trait anxiety exhibit greater activation of the Broca's area when performing inhibitory control tasks. This activity may represent an adaptive mechanism that supports top-down control. When confronted with threatening words, individuals with higher anxiety levels demonstrated increased left prefrontal activity in the Broca's area [28]. This suggests that Broca's area is crucial in processing emotional stimuli.

### 1.5. Study Rationale and Objectives

Given that music therapy is a commonly used intervention for mental health, this study employed music therapy to treat individuals with anxiety and utilized fNIRS to assess its neural effects. To the best of our knowledge, this is the first study to apply fNIRS to evaluate the impact of music therapy on anxiety. The primary objective of this study was to use fNIRS to quantitatively assess the effects of music therapy on anxiety symptoms and brain network activity across different treatment durations. The secondary objective was to explore the clinical potential of fNIRS-derived biomarkers.

This study defined short-term intervention as the immediate effects following a single 15-min music therapy session, assessed within 30 min postintervention. Long-term intervention referred to the cumulative effects of 12 sessions administered over 4 weeks, evaluated 24 h after the final session. The aim was to differentiate acute neuromodulation from sustained neuroplastic adaptations.

## 2. Methods

### 2.1. Overall Flowchart

**Figure 1:**
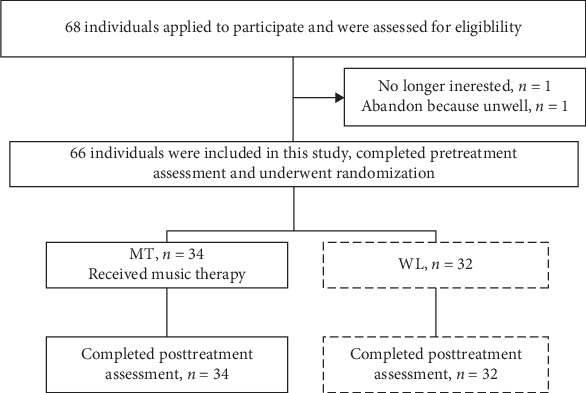
Schematic diagram of the experimental procedure. Note: MT, music therapy group; WL, wait-list controls group.

This study employed a single-masked, randomized, controlled experimental design. This study employed a single-blind design. Specifically, the research assistants who administered the psychological scales (GAD-7 and PHQ-9) and the technicians who processed and analyzed the fNIRS data were blinded to the group assignment (intervention or waiting list) of the participants. However, the participants themselves and the music therapist conducting the sessions were not blinded to the intervention due to the inherent nature of the therapy. This study was conducted between October 2023 and December 2023 at the Psychology Laboratory of the First Clinical College of Hainan Medical University.  Figure 1 shows the experimental flowchart. Twelve music therapy intervention sessions were conducted, with evaluations conducted after the first and twelfth sessions. The specific content and processes of the music therapy intervention are presented in Table 1.

### 2.2. Participants

All participants were recruited from Hainan Medical University. A total of 83 eligible participants aged 17–21 years were selected through an advertisement screening process. Among them, 66 were assigned to the anxiety group and randomly divided into two subgroups (intervention group: 34 participants; waiting group: 32 participants). The randomization was performed using a computer-generated random number sequence (created in SPSS v26.0 by the “Transform ⟶ Random Number Generators ⟶ Set Starting Point” and “Data ⟶ Select Cases ⟶ Random sample” functions) by an independent researcher who was not involved in participant recruitment, assessment, or the intervention process. The group allocation was concealed from the participants until after the baseline (T1) assessment was completed. Additionally, 17 participants were enrolled in the matched healthy control group (Health Control group, HC).

All participants were current undergraduate students who met the inclusion and exclusion criteria. All participants fully understood the study's contents, voluntarily participated, and signed informed consent forms. The inclusion criteria for the groups were as follows: for the healthy control group, a GAD-7 total score < 5; for the anxiety group, a GAD-7 total score ≥5, indicating at least mild anxiety symptoms [29]. The exclusion criteria for all participants were (1) history of mental illness (e.g., depression and schizophrenia), (2) neurological disorders (e.g., epilepsy and traumatic brain injury), screened with reference to the contents of the Neurological Disorders Screening Form (NDSF-5), (3) suicidal ideation/behavior, (4) use of psychotropic medications, and (5) substance abuse history.

It is important to note that participants were recruited from a university student population based on self-reported symptom scales and did not undergo a formal clinical diagnosis by a psychiatrist. Therefore, they are best characterized as individuals with elevated anxiety symptoms rather than clinically diagnosed anxiety disorder.

### 2.3. Human Ethics and Consent to Participate Declarations

The Ethics Committee of the First Affiliated Hospital of Hainan Medical University approved this study (Approval Number 2024-KYL-133). All participants were informed about the content and interventions of the experiment and provided their consent. This study adheres to the ethical principles for human research outlined in the Declaration of Helsinki.

### 2.4. Equipment Information

#### 2.4.1. Equipment Parameter Information

The experiment utilized the NirSmart (NirSmartⅡ-3000A) system (Danyang Huichuang Medical Equipment Co., Ltd., Jiangsu, China), which has been applied in previous studies [30, 31]. This device continuously measures and records changes in the concentrations of oxygenated hemoglobin (HbO) and deoxygenated hemoglobin (HbR) in the resting-state brains of the participants. The system comprises a near-infrared light source (light-emitting diode, LED) and avalanche photodiode (APD) detectors. The light source probes were operated at 730 and 850 nm wavelengths, with a sampling frequency of 11 Hz.

#### 2.4.2. Probe Arrangement and Brain Regions Covered

**Figure 2:**
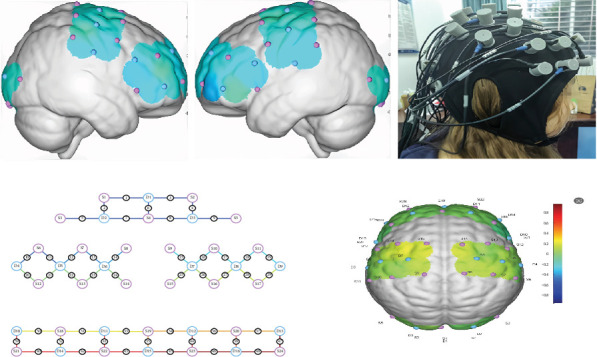
fNIRS probe configuration and channel localization. (A) Layout of the fNIRS optodes and channels on the scalp. Red dots represent the 24 source probes, blue dots represent the 16 detector probes, and green lines represent the 48 measurement channels. The optodes were positioned to cover the prefrontal, frontal, parietal, occipital, and temporal lobes based on the international 10–20 system. (B) Projection of the fNIRS channel coordinates onto the Montreal Neurological Institute (MNI) standard brain template. The coordinates for each channel were derived from the probe layout in (A) and spatially registered using the method provided by the NirSpace software. Each dot represents the estimated location of a channel, color-coded according to its corresponding Brodmann area.

Adjacent near-infrared light sources and detectors were arranged at 3 cm intervals. The connection between adjacent light sources and detectors is defined as a “channel.” In this experiment, the 48 channels of the device consisted of 24 light source probes and 16 detector probes, covering the entire brain region of the frontal, parietal, occipital, and temporal lobes, based on Brodmann area localization (Figure 2A).

#### 2.4.3. Channel Coordinate Acquisition

Following the standard international 10/20 electrode placement system, the obtained coordinates were converted into MINI coordinates and projected onto the MINI standard brain template using the spatial registration method provided by NirSpace (Danyang Huichuang Medical Equipment Co., Ltd., Jiangsu, China) (Figure 2B).

### 2.5. Music Therapy Intervention and Assessment Procedure

Music interventions have demonstrated significant stress-reducing effects on psychological stress (e.g., state anxiety) and physiological indices (e.g., heart rate, blood pressure, and hormones). Notably, these effects are often immediate, achievable even with single-session, short-duration interventions [32, 33]. Studies have also shown that online group music therapy has a significant alleviating effect on anxiety among university students [34]. Based on the aforementioned evidence and clinical practice, the present protocol combined music-assisted relaxation with guided imagery and was administered in short sessions (8–15 min each).

The music therapy intervention comprised 12 modules (detailed in Table 1), with every four modules forming one thematic unit. Three modules were administered per week, resulting in completion of the entire intervention within 4 weeks. Each module, lasting between 8 and15 min, involved listening to soft background music accompanied by slow and soothing relaxation training instructions incorporating guided imagery and mindfulness techniques to facilitate self-relaxation. Crucially, fNIRS data were acquired concurrently during these music therapy sessions. The assessment procedure involved baseline assessment (T1) prior to any intervention, where all participants from both the intervention and waiting-list groups completed self-report psychological scales (GAD-7 and PHQ-9), followed by participation in a music therapy session while undergoing fNIRS recording to establish preintervention baseline measures of brain activity in response to musical stimulus. The postintervention assessment (T2) was conducted after the intervention group completed their 12th and final music therapy session, during which all participants again completed the psychological scales and underwent a final fNIRS recording during an identical music therapy session. For the intervention group, this session represented their 12th therapeutic session, while for the waiting-list group, it served as their preintervention baseline before subsequently receiving the therapy. All assessments were conducted in a quiet, controlled laboratory environment, with each assessment time point (T1 and T2) requiring ~30–40 min to complete.

Each individual therapy session was conducted under a standardized setting and procedure, as detailed below: The music therapy interventions were conducted in a dedicated, sound-attenuated room within the Psychology Laboratory of the First Clinical College of Hainan Medical University. The environment was designed to minimize distractions and promote relaxation, featuring softly dimmed lighting and a maintained comfortable temperature of ~22–24°C, with participants seated in a comfortable reclining chair throughout each session. The intervention protocol was adapted from established music therapy techniques for anxiety management that emphasize receptive music listening combined with guided relaxation and imagery, while the specific content of the 12 modules (Table 1) incorporated principles from mindfulness-based stress reduction and guided imagery. Each session followed a standardized procedure beginning with a 2-min preparation phase where the researcher greeted the participant, ensured their comfort, and fitted the fNIRS headcap while verifying audio equipment functionality. This was followed by the 8–15 min intervention phase during which participants were instructed to close their eyes, relax, and focus attention on the prerecorded audio program delivered through high-quality noise-canceling headphones; the program seamlessly integrated soft instrumental music characterized by slow tempos, low pitch, and harmonious melodies with spoken guided instructions for relaxation, breathwork, and imagery such as visualization of peaceful natural scenes. The session concluded with a 1-min completion phase where participants were given moments of silence after the audio program ended before the researcher gently instructed them to open their eyes and end the session.

### 2.6. Data Processing and Statistical Methods

#### 2.6.1. Data Preprocessing

Following data acquisition, fNIRS signals underwent preprocessing via NIRspark (v1.8.1; Huichuang Medical Equipment Co., China) implementing sequential quality control initiating with participant-level exclusion based on <80% valid signal channels, followed by automated channel stability assessment through coefficient of variation (CV) analysis using DataQualityAnalysisTool.exe (Huichuang Medical) with classification thresholds (CV ≤ 5%: optimal; 5% < CV ≤ 20%: borderline requiring verification; CV >20%: automatically excluded), proceeding to motion artifact correction (std = 6, am*p*=0.5) and physiological noise filtering (bandpass 0.01–0.2 Hz); subsequent quality assurance incorporated targeted visual inspection specifically focused on borderline channels (5% < CV ≤ 20%) to identify nonfluctuating artifacts including signal saturation, flatlining or uncorrected drifts, alongside truncation of unstable onset/offset segments (initial/final 10–30 s), culminating in conversion of denoised optical density data to HbO concentrations via the modified Beer–Lambert law, where FC analysis was exclusively restricted to dual-validated channels meeting both CV ≤ 20% criteria and artifact-free confirmation.

#### 2.6.2. Data Analysis

This experiment used NirSpark analysis software (V1.8.1) to process and analyze the raw fNIRS data. The software was implemented in MATLAB (MathWorks, USA). Data analysis primarily focused on brain network analysis, including Fisher's R-to-Z transformations, difference testing, correlation analysis, and multiple comparison corrections, all performed using NirSpark analysis software (V1.8.1).

In addition, SPSS (V26.0) was used to conduct chi-square analyses and independent-sample *t*-tests on the preintervention data of the three participant groups. Paired-sample *t*-tests were also performed to evaluate changes in anxiety scores before and after the intervention.

For all channel-pair connectivity analyses, false discovery rate (FDR) correction was applied using the Benjamini–Hochberg procedure implemented in NirSpark software (v1.8.1). The significance threshold was set at *q* < 0.05, controlling the expected proportion of false positives to ≤5%.

#### 2.6.3. Statistical Hypothesis Testing

Specific statistical tests were applied to evaluate hypotheses concerning FC data. Between-group comparisons of global mean FC at baseline were conducted using the Kruskal–Wallis *H* test due to violations of normality assumptions. Significant main effects were further explored with post hoc pairwise comparisons using the Dunn–Bonferroni method. Within-group comparisons (pre- versus postintervention) for FC were performed using paired *t*-tests for normally distributed data and nonparametric Wilcoxon signed-rank tests for nonnormally distributed data, as was the case for the analysis immediately following the first intervention session. The FDR method (Benjamini–Hochberg procedure) was applied to correct for multiple comparisons across all channel pairs in the whole-brain connectivity analysis.

#### 2.6.4. FC and Network Analysis

FC was computed at the channel level based on pairwise correlations of the preprocessed HbO time series between all 48 channels. This generated a full, weighted 48 × 48 connectivity matrix for each participant. The global mean FC, reported as a summary metric, was calculated as the average of all correlation coefficients in this matrix, representing the overall strength of functional integration across the entire brain network. Although channels were mapped to anatomical regions (Figure 2B) for interpretation, all statistical comparisons were performed on channel-pair connectivity without regional pooling to maintain the spatial specificity of the analysis.

For graph-theoretical network analysis, a sparsity threshold of 30% was applied to each participant's connectivity matrix to create a binarized network. This threshold retains only the top 30% strongest connections (i.e., the 338 strongest edges), thereby controlling for inter-individual differences in overall connection strength and allowing for the analysis of network topology independent of absolute correlation values.

## 3. Results

### 3.1. Demographic and Assessment Results

The results of the difference tests for the demographic and psychological assessments are presented in Table 2. No significant differences were found between the intervention, waiting, and healthy groups regarding age, sex, or years of education. The anxiety score results indicated significant differences among the three groups, with post hoc tests showing that the anxiety scores of the healthy group were significantly lower than those of the intervention and waiting groups.

### 3.2. Baseline Brain Network Characteristics Based on Channels Before Intervention

#### 3.2.1. Before Intervention

**Figure 3:**
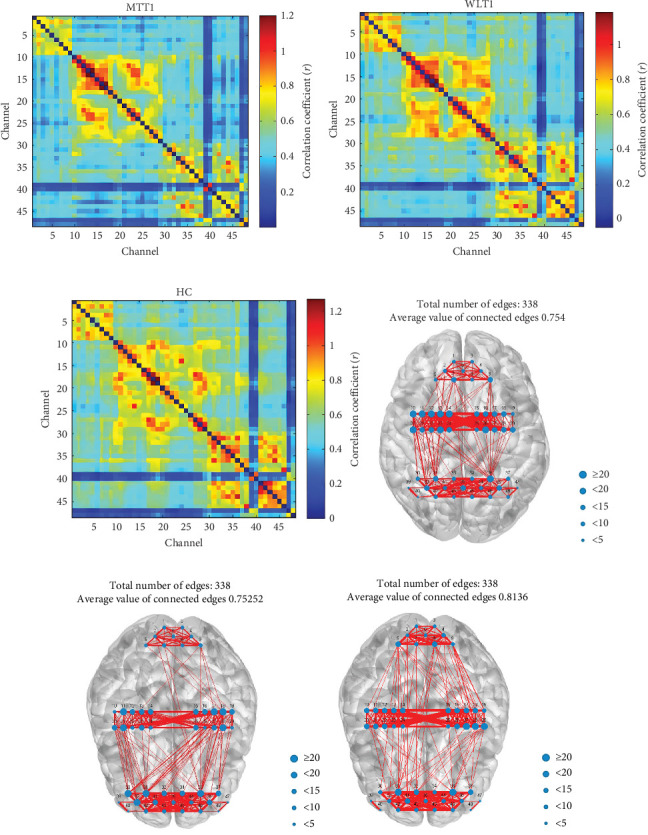
Baseline functional connectivity and network topology differ between groups. (A–C) Channel-wise functional connectivity matrices (Pearson's correlation) for the (A) music therapy (MT, *N* = 34), (B) waiting list (WL, *N* = 32), and (C) healthy control (HC, *N* = 17) groups at baseline (T1). Both the *x*-axis and *y*-axis represent the 48 fNIRS channels. The color bar indicates the strength of the correlation coefficient from −1 to 1. (D–F) Binary adjacency matrices representing the top 30% strongest connections (sparsity threshold = 0.3) for the (D) MT, (E) WL, and (F) HC groups. The average network edge weight for each group is displayed above each matrix. Statistical analysis: a Kruskal–Wallis test revealed a significant difference in global mean functional connectivity among the three groups (*H* = 36.67, *p* < 0.001). Post hoc Dunn–Bonferroni pairwise comparisons indicated that the healthy control group had significantly higher functional connectivity than both the music therapy group (*p* < 0.001) and the waiting-list group (*p* < 0.001), with no significant difference between the two anxiety groups.

The experimental results showed that the mean FC was 0.437 ± 0.141 in the intervention group (*N* = 34), 0.448 ± 0.153 in the waiting group (*N* = 32), and 0.501 ± 0.158 in the healthy control group (*N* = 17). Channel-wise brain network connectivity heat maps are shown in Figure 3A–C. A Kruskal–Wallis test revealed a significant difference in mean FC among the three groups (*H* = 36.67, *p*  < 0.001). Post hoc Dunn–Bonferroni pairwise comparisons indicated higher FC in healthy controls versus the intervention and waiting groups, with no significant difference between the intervention and waiting groups (Table 3).

To control for inter-individual differences in overall connection strength and analyze network topology, we applied a sparsity threshold of 0.31. This analysis revealed that the average weight of the top 30% of connections was 0.754, 0.752, and 0.814 for the MT, WL, and HC groups, respectively (Figure 3D–F).

### 3.3. Result Analysis After the First Intervention (T1)

**Figure 4:**
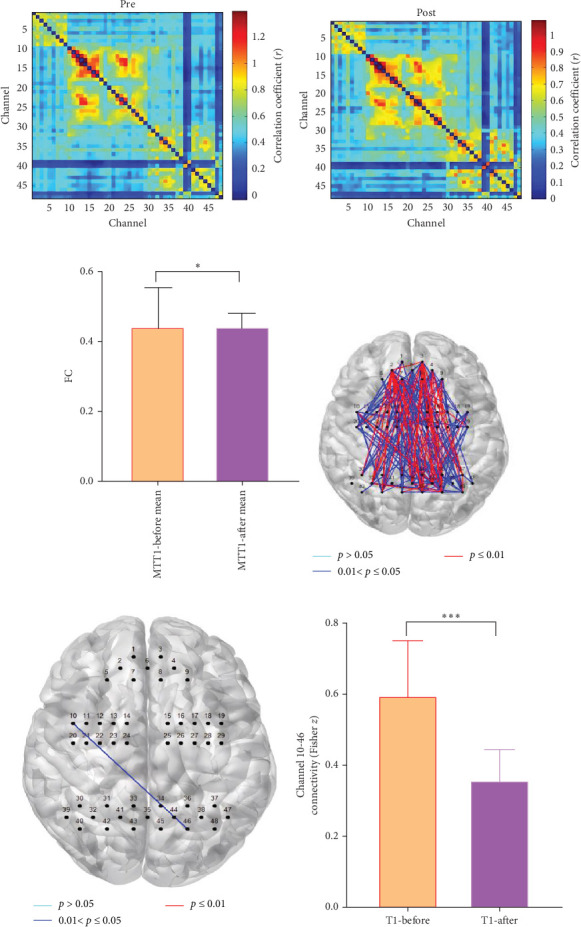
Immediate reduction of functional connectivity following a single music therapy session. (A, B) Global mean functional connectivity matrices (±SD) in the music therapy (MT, *N* = 34) group (A) before (pre) and (B) after (post) the first intervention session. Both axes represent the 48 channels. (C) The change in global mean functional connectivity (post–pre) for the MT group. A Wilcoxon signed-rank test revealed a significant reduction (*p*^⁣^*∗*^^=0.017). (D, E) Connectivity values for the significant channel pair 10–46 (linking the supramarginal gyrus/Wernicke's area and the dorsolateral prefrontal cortex) (D) before and (E) after the intervention. (F) The significant reduction in connectivity strength for channel pair 10–46 following the intervention (*⁣*^*∗∗∗*^*p* < 0.001, Wilcoxon signed-rank test). Data are presented as mean ± SD. ⁣^*∗*^*p*^⁣^*∗*^^ < 0.05, ⁣^*∗∗*^*p*^⁣^*∗*^^ < 0.01, ⁣^*∗∗∗*^*p*^⁣^*∗*^^ < 0.001.

After one intervention session, the mean network connectivity values for each channel in the intervention group were analyzed before and after the first music therapy session. The mean FC decreased from 0.437 ± 0.141 preintervention (Figure 4A) to 0.387 ± 0.159 immediately postintervention (Figure 4B). A nonparametric Wilcoxon signed-rank test revealed a significant reduction in global mean FC between the two time points (*Z* = 2.486, *p*=0.017) (Figure 4C).

Using a sparsity threshold of 0.3 [35], 338 network edges across 48 channels were analyzed. Before FDR correction, paired *t*-tests revealed significant pre-to-post reductions in FC at 64 channel pairs (*p*  < 0.05), as visualized in Figure 4D. After FDR correction (Benjamini–Hochberg procedure, *q* < 0.05), only one channel pair (10–46), linking the supramarginal gyrus/Wernicke's area and the DLPFC, retained significance (Figure 4E). The connectivity values of channel 10–46 in the intervention group significantly decreased from 0.602 ± 0.188 before the intervention (T1), which is a notably high value for a single connection compared to the group's global network average (0.754), to 0.366 ± 0.302 after the intervention. A Wilcoxon signed-rank test confirmed this significant reduction (*Z* = 3.680, *p* < 0.001; Figure 4F).

### 3.4. Results After 12 Interventions (T2)

At baseline, no significant differences existed in GAD-7 or PHQ-9 scores between intervention and waiting groups (GAD-7: *t* (66) = 0.20, *p*=0.842; PHQ-9: *t* (66) = 1.12, *p*=0.267). After 12 sessions, the intervention group showed significant reductions in both GAD-7 (*Δ* = −1.97 ± 2.15, *t* [33] = 5.18, *p* < 0.001) and PHQ-9 scores (*Δ* = −4.19 ± 3.62, *t* [33] = 6.55, *p*  < 0.001), whereas the waiting group exhibited no significant changes (GAD-7: *Δ* = 0.72 ± 4.60, *t* [31] = 0.94, *p*=0.355; PHQ-9: *Δ* = −1.28 ± 2.87, *t* [31] = 1.30, *p*=0.203). These results indicated that music therapy has a noticeable effect on anxiety reduction.

**Figure 5:**
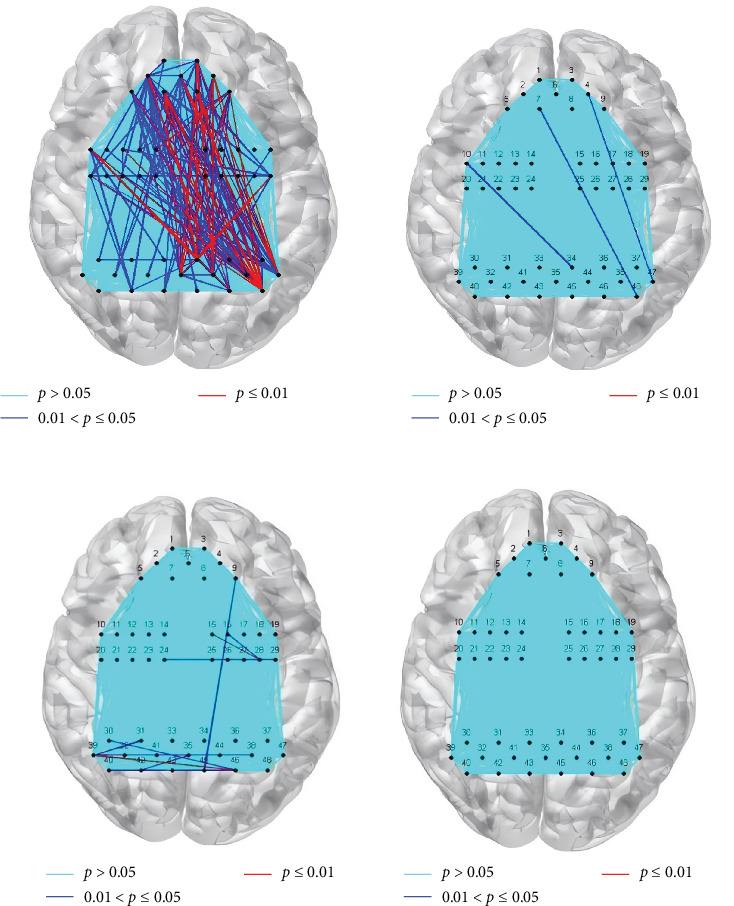
Longitudinal changes in functional connectivity after 12 sessions of music therapy. (A) Within the music therapy group (MT, *N* = 34), 28 channel pairs showed significant reductions in functional connectivity from pre- to postintervention before false discovery rate (FDR) correction (paired *t*-tests, *p* < 0.05). (B) After FDR correction (Benjamini–Hochberg procedure, *q* < 0.05), three channel pairs retained significance in the MT group: Channel 10–34 (Broca's pars opercularis—dorsolateral prefrontal cortex); Channel 7–48 (primary visual cortex V1—Broca's pars triangularis); Channel 4–47 (visual association cortex V2—Broca's pars triangularis). (C) In the waiting-list group (WL, *N* = 32), 10 channel pairs showed nominally significant within-group longitudinal changes before FDR correction (paired *t*-tests, *p* < 0.05). (D) After FDR correction, no channel pairs in the WL group retained significance, indicating no reliable changes in functional connectivity over time. Note: FDR correction was applied to control for multiple comparisons. Connectivity values are based on pairwise correlations of oxygenated hemoglobin (HbO) time series.

After completing 12 interventions, within-intervention-group longitudinal comparisons revealed significant pre-to-post reductions in FC at 28 channel pairs before FDR correction (paired *t*-tests: *p* < 0.05), as visualized in Figure 5A. After FDR correction (using Benjamini–Hochberg procedure at *q* < 0.05), three channel pairs retained significance (paired *t*-tests: *p* < 0.05), as visualized in Figure 5B. The network connectivity of the following channels showed a significant reduction:

  Channels 10–34 (pars opercularis, part of Broca's area, and DLPFC): connectivity weakened from 0.601 ± 0.263 to 0.299 ± 0.271 (*t* = 4.651, FDR-corrected *q* = 0.038).
  Channels 7–48 (primary visual cortex [V1]—pars triangularis of Broca's area): connectivity significantly decreased from 0.357 ± 0.273 to 0.029 ± 0.172 (*t* = 4.693, FDR-corrected *q* = 0.038).
  Channel 4–47 (visual association cortex [V2]—pars triangularis of Broca's area): connectivity significantly reduced from 0.267 ± 0.291 to −0.080 ± 0.234 (*t* = 4.855, FDR-corrected *q* = 0.037).

These results indicate significant differences in channel connectivity after FDR correction. In contrast, the waiting group exhibited nominally significant within-group longitudinal changes in FC for 10 channel pairs before FDR correction (paired *t*-tests: 0.01 < *p*  < 0.05), visualized in Figure 5C. However, none survived FDR correction (Benjamini–Hochberg, *q* < 0.05), indicating no reliable changes over time (Figure 5D).

### 3.5. Correlation Between Changes in Anxiety Symptoms and Brain Connectivity

To directly investigate the relationship between the therapeutic effect of music therapy and modulation of brain networks, we performed Spearman's correlation analyses between the improvement in anxiety symptoms (*Δ*GAD-7) and the changes in FC (*Δ*FC) within the music therapy group for the three key channels that showed significant intervention effects. As shown in Table 4, we found statistically significant negative correlations for channel 10–34 (linking Wernicke's area and the DLPFC; *ρ*=−0.478, *p*=0.008) and channel 4–47 (linking the visual association cortex and Broca's area; *ρ*=−0.394, *p*=0.032). The correlation for channel 7–48 (linking the primary visual cortex and Broca's area) was not significant (*ρ*=−0.241, *p*=0.200). These results indicate that greater reduction in anxiety symptoms was associated with greater reduction in FC in these specific higher-order cognitive and associative pathways.

## 4. Discussion

The primary aim of this study was to investigate the neuromodulatory effects of music therapy on anxiety symptoms and brain FC using fNIRS. Our findings can be summarized in two key aspects: (1) behaviorally, a 12-session online music therapy protocol significantly reduced anxiety and depressive symptoms in university students compared to a waitlist control, corroborating previous meta-analyses [36, 37]. (2) Neurophysiologically, we found that music therapy normalized aberrant brain connectivity by reducing hyperactivity in specific prefrontal-limbic-temporal circuits, and these reductions were significantly correlated with clinical improvement.

### 4.1. Neural Effects of Music Therapy: Immediate and Long-Term Changes

Our fNIRS data provide novel evidence for the neural impact of music therapy. After just a single session, we observed a significant immediate reduction in global mean FC. More importantly, a specific hyperconnected pathway between the supramarginal gyrus (part of Wernicke's area) and the DLPFC (channel 10–46) was markedly weakened. This suggests that music therapy can induce rapid neurophysiological changes, potentially initiating a process of normalizing maladaptive brain network activity.

The long-term effects after 12 sessions were more pronounced and targeted. The intervention group showed significant symptom improvement, which was paralleled by a refined reduction in FC within several critical circuits after FDR correction. These included connections between Broca's pars opercularis and the DLPFC (channel 10–34), the primary visual cortex (V1) and Broca's pars triangularis (channel 7–48), and the visual association cortex (V2) and Broca's pars triangularis (channel 4–47). In contrast, the waiting group showed no significant changes in either symptoms or FC after correction. This convergence of behavioral and neural data strongly indicates that the alleviation of anxiety symptoms is associated with the modulation of FC in a network involving prefrontal, language, and visual processing regions.

Our study also directly addressed the relationship between neural changes and symptom improvement. The significant negative correlations we observed between anxiety reduction (*Δ*GAD-7) and reduced connectivity in channels 10–34 (*ρ*=−0.478, *p*=0.008) and 4–47 (*ρ*=−0.394, *p*=0.032) provide crucial support for a mechanistic account of music therapy. This finding suggests that the clinical benefits are not merely concomitant with brain changes but are specifically linked to the normalization of FC within networks encompassing the DLPFC (a hub for cognitive control), Wernicke's area (language comprehension), and the visual association cortex (emotional imagery). The negative direction of the correlation implies that music therapy may alleviate anxiety by downregulating hyperconnected pathways that might contribute to maladaptive cognitive processes (e.g., rumination and catastrophic thinking). The lack of a significant correlation for channel 7–48, which involves the primary visual cortex, further strengthens this interpretation by demonstrating the specificity of this effect to higher-order associative and cognitive brain networks, rather than primary sensory areas. Therefore, these results move beyond correlation and suggest that modulating FC in these specific circuits is a plausible mechanism through which music therapy exerts its therapeutic effects.

### 4.2. Interpretation of the FC Decrease

A seemingly paradoxical yet critical finding of our study is the direction of FC changes following effective music therapy. Contrary to a simplistic expectation that therapy might “normalize” FC by elevating it to healthy control levels, we observed a further reduction in FC within several specific, hyperconnected pathways in the anxiety group.

We interpret this localized decrease not as a deleterious effect but as a potential biomarker of successful network refinement. The pretreatment hyperconnectivity in these specific circuits may reflect a state of maladaptive compensation, a known feature of anxiety symptoms where neural networks engage in excessive, effortful, yet inefficient attempts to regulate heightened emotional arousal [38]. This is consistent with the broader neurobiological principle that psychopathology is often associated with a deviation from the optimal, efficient small-world network architecture that characterizes healthy brain function [39].

We posit that music therapy, by promoting relaxation and altering emotional processing, ameliorates the core drivers of this hyperarousal. Consequently, the need for such energetically costly compensatory overactivation is reduced. The subsequent down-regulation or “pruning” of FC in these hyperconnected pathways signifies a transition toward a more metabolically efficient and specialized neural state. This normalization process may occur through the enhancement of targeted, top-down inhibitory control mechanisms mediated by the prefrontal cortex, which are crucial for regulating emotional responses [40]. Thus, the reduction in FC within these specific circuits is a testament to the therapy's efficacy in restoring a more adaptive and efficient neural equilibrium, moving the network configuration closer to the optimal small-world organization.

### 4.3. Implications of Key Brain Regions

The brain regions identified in our study align with the neuropathology of anxiety. The DLPFC is a core hub for executive control and cognitive regulation [41]. Its dysfunctional engagement is a hallmark of anxiety symptoms. The involvement of Broca's area (pars triangularis and opercularis) and Wernicke's area is particularly intriguing. These regions are not only central to language processing but are also critically involved in the interpretation of social cues, internal dialog, and the cognitive appraisal of threatening stimuli. Their hyperconnectivity with prefrontal and visual cortices in our anxiety groups may represent a neural correlate of ruminative thinking, negative self-talk, and heightened vigilance. Music therapy, by providing a structured, nonverbal emotional stimulus, may directly modulate this network, facilitating a shift from maladaptive cognitive–evaluative processes to more direct emotional processing and regulation.

### 4.4. Potential of fNIRS-Based Biomarkers for Anxiety

Beyond demonstrating the efficacy of music therapy, our findings regarding the specific dysregulated pathways (e.g., hyperconnectivity in channels 10–34, 7–48, and 4–47) hold significant potential as objective fNIRS-based biomarkers for anxiety. These biomarkers could not only aid in the auxiliary diagnosis of anxiety by providing a neurophysiological correlate to subjective symptoms but could also serve as targets for novel neuromodulation therapies (e.g., neurofeedback) and provide a quantifiable means to evaluate treatment response.

### 4.5. Conclusion and Clinical Relevance

In conclusion, this study demonstrates that music therapy effectively alleviates anxiety symptoms and normalizes aberrant functional brain connectivity. We provide direct fNIRS evidence that its effects are mediated by the refinement of hyperconnected neural circuits involving the prefrontal cortex, Broca's area, and Wernicke's area, reducing inefficient compensatory activity and promoting more optimal network function.

Furthermore, our findings suggest that fNIRS-derived FC metrics, particularly in the identified channels, could serve as objective biomarkers for the auxiliary diagnosis and efficacy evaluation of anxiety. This study underscores the value of music therapy as a nonpharmacological intervention and highlights the potential of fNIRS as a tool for unveiling the neural mechanisms of psychological therapies.

## 5. Limitations

Several limitations should be considered. First, our participants were recruited from a nonclinical student population based on self-report scales. While they exhibited elevated anxiety symptoms, they were not formally diagnosed with an anxiety disorder. This may limit the generalizability of our findings to clinical populations with diagnosed anxiety symptoms. Future studies should replicate these findings in clinically diagnosed samples.

Second, this study primarily collected the resting-state fNIRS data. Compared to resting-state data, task-state data may be more effective in identifying anxiety. Therefore, the next step was to build upon this experiment by incorporating task-state data and combining both resting-state and task-state analyses to improve the identification of anxiety.

Finally, while we collected data on key clinical exclusions (medication use and substance abuse) and comorbid depressive symptoms, we did not record other demographic variables such as body mass index (BMI) or lifestyle factors (e.g., caffeine intake). Future studies could include these measures to further rule out their potential confounding effects and to provide a more comprehensive characterization of the sample.

## 6. Conclusion

### 6.1. The Immediate and Cumulative Effects of Music Therapy

Compared to the existing literature, this study conducted a longitudinal follow-up, revealing that changes in brain network connectivity occurred to varying degrees after one session and 12 sessions of music therapy. This indicated that music therapy has significant immediate and cumulative effects over time. This suggests that with an increasing number of therapy sessions, the regulatory effect of music therapy on brain networks gradually became more pronounced and enhanced.

### 6.2. Brain Regions Associated With Anxiety

After completing 12 intervention sessions and applying FDR correction, the network connectivity in the brain of the intervention group showed significant reductions in the following channels: Wernicke's area and DLPFC (channels 10–34), primary visual cortex and pars triangularis of Broca's area (channels 7–48), and visual association cortex and pars triangularis of Broca's area (channel 4–47). These significant differences in channel connectivity indicate that anxiety symptoms are related to these brain regions. Furthermore, our study suggests that fNIRS-derived FC metrics, particularly in the identified channels (e.g., 10–34, 7–48, and 4–47), can serve as objective biomarkers for the auxiliary diagnosis and efficacy evaluation of anxiety.

## Figures and Tables

**Table 1:** Music therapy protocol.

Content	Time	Content	Time	Contents	Time	Contents	Time
Breathing and muscle relaxation	10 min	Empty the box	9 min	Nature meditation relaxation	14 min	My safe cabin	9 min
Put down the backpack	12 min	Seaside visualization	11 min	The power of tides	8 min	Inner wisdom	13 min
Satir meditation	10 min	Nature visualization	13 min	Listening to the self	8 min	Inner safe island	15 min

**Table 2:** Results of difference tests for demographics and psychological assessments.

Variables	MT(*N* = 34)	WL (*N* = 32)	HC (*N* = 17)	Statistic	Post hoc test
Sex (male/female)	8/26	5/27	4/13	*χ^2^* = 1.54	
Age (years)	18.54 ± 0.69	18.31 ± 0.82	18.73 ± 0.09	*F* = 2.43	
Education (years)	13.20 ± 0.62	13.06 ± 0.26	13.08 ± 0.29	*F* = 0.86	
GAD-7 T1	7.21 ± 4.74	7.00 ± 4.02	2.50 ± 1.47	*H* = 9.74*⁣*^*∗∗∗*^	MT > HC,WL > HC
PHQ-9 T1	10.03 ± 3.84	11.15 ± 4.30	3.20 ± 1.98	*H* = 30.70*⁣*^*∗∗∗*^	MT > HC,WL > HC
GAD-7 T2	5.24 ± 4.81	7.72 ± 3.99		*H* = 2.45*⁣*^*∗∗∗*^	
PHQ-9 T2	5.84 ± 4.58	9.87 ± 5.32		*H* = 3.58*⁣*^*∗*^	

*Note:* T1: before intervention; T2: after completing 12 Interventions. Data are presented as mean ± standard deviation or number of participants. Statistical analysis: for continuous variables, one-way ANOVA was used for age and education (normally distributed), and the Kruskal–Wallis *H* test was used for GAD-7 and PHQ-9 scores (nonnormally distributed). HC, healthy control group; MT, music therapy group; WL, waiting-list group.

Abbreviations: GAD-7, generalized anxiety disorder-7; PHQ-9, patient health questionnaire-9.

*⁣* ^*∗*^ *p* < 0.05.

*⁣* ^*∗∗*^ *p* < 0.01.

*⁣* ^*∗∗∗*^ *p* < 0.001.

**Table 3:** Comparison of differences in mean FC based on channels among the three groups.

Group	*n*	FC (mean ± SD)	FC (sparsity = 0.3) (mean ± SD)	Statistical value	*p*	Post hoc
MTT1	34	0.437 ± 0.141	0.754 ± 0.176			a
WLT1	32	0.448 ± 0.153	0.752 ± 0.123			a
HC	17	0.501 ± 0.168	0.814 ± 0.157			b
Test statistic		–	–	*H* = 36.67	<0.001	

*Note:* Statistical analysis by Kruskal–Wallis test due to nonnormal distribution of functional connectivity. Values are presented as mean ± standard deviation or median (interquartile range). Statistical analysis: a Kruskal–Wallis *H* test revealed a significant difference among groups (*H* = 36.67, *p* < 0.001). Post hoc pairwise comparisons were conducted using Dunn's test with a Bonferroni correction. Groups with different superscript letters (a, b) are significantly different at *p* < 0.05. HC, healthy control group; MT, music therapy group; WL, waiting-list group.

**Table 4:** Spearman's correlations between improvement in anxiety symptoms (*Δ*GAD-7) and changes in functional connectivity (*Δ*FC) after music therapy.

Channel	Approximate brain regions	Spearman's *ρ*	*p*-Value	Significance (*α* = 0.05)
10–34	Wernicke's area—DLPFC	**−0.478**	**0.008**	Significant
7–48	Primary visual cortex—Broca's area	−0.241	0.200	Not significant
4–47	Visual association cortex—Broca's area	**−0.394**	**0.032**	Significant

*Note: Δ*GAD-7 = GAD-7 score at T2—GAD-7 score at T1; *Δ*FC = functional connectivity value at T2—FC value at T1. A negative correlation indicates that a greater reduction in anxiety symptoms is associated with a greater reduction in functional connectivity. The bold is used to highlight the statistically significant results (*p*-value < 0.05).

## Data Availability

The data that support the findings of this study are available from the corresponding author, Lijuan Liang, upon reasonable request.

## Nomenclature

APD: Avalanche photodiode
CBT: Cognitive behavioral therapy
DLPFC: Dorsolateral prefrontal cortex
FC: Functional connectivity
FDR: False discovery rate
fNIRS: Functional near-infrared spectroscopy
GAD: Generalized anxiety disorder
HbO: Oxygenated hemoglobin
HbR: Deoxygenated hemoglobin
LED: Light-emitting diode
MDD: Major depressive disorder
NIRS: Near-infrared spectroscopy
PD: Panic disorder
RCTs: Randomized controlled trials
rTMS: Repetitive transcranial magnetic stimulation
SAD: Social anxiety disorder
SSRIs: Selective serotonin reuptake inhibitors
SNRIs: Serotonin-norepinephrine reuptake inhibitors.
